# Ultrasound-based deep learning radiomics model for differentiating benign, borderline, and malignant ovarian tumours: a multi-class classification exploratory study

**DOI:** 10.1186/s12880-024-01251-2

**Published:** 2024-04-15

**Authors:** Yangchun Du, Wenwen Guo, Yanju Xiao, Haining Chen, Jinxiu Yao, Ji Wu

**Affiliations:** 1 Department of Ultrasound, The First Affiliated Hospital of Guangxi Medical University, No.6 Shuangyong Road, Qingxiu District, 530021 Nanning, China; 2 Department of Ultrasound, The People’s Hospital of Guangxi Zhuang Autonomous Region & Guangxi Academy of Medical Sciences, No.6 Taoyuan Road, Qingxiu District, 530021 Nanning, China; 3 Department of Pathology, The People’s Hospital of Guangxi Zhuang Autonomous Region & Guangxi Academy of Medical Sciences, No.6 Taoyuan Road, Qingxiu District, 530021 Nanning, China

**Keywords:** Ovarian tumour, Ultrasound, Hand-crafted radiomics, Deep transfer learning signature, Multi-class classification model

## Abstract

**Background:**

Accurate preoperative identification of ovarian tumour subtypes is imperative for patients as it enables physicians to custom-tailor precise and individualized management strategies. So, we have developed an ultrasound (US)-based multiclass prediction algorithm for differentiating between benign, borderline, and malignant ovarian tumours.

**Methods:**

We randomised data from 849 patients with ovarian tumours into training and testing sets in a ratio of 8:2. The regions of interest on the US images were segmented and handcrafted radiomics features were extracted and screened. We applied the one-versus-rest method in multiclass classification. We inputted the best features into machine learning (ML) models and constructed a radiomic signature (Rad_Sig). US images of the maximum trimmed ovarian tumour sections were inputted into a pre-trained convolutional neural network (CNN) model. After internal enhancement and complex algorithms, each sample’s predicted probability, known as the deep transfer learning signature (DTL_Sig), was generated. Clinical baseline data were analysed. Statistically significant clinical parameters and US semantic features in the training set were used to construct clinical signatures (Clinic_Sig). The prediction results of Rad_Sig, DTL_Sig, and Clinic_Sig for each sample were fused as new feature sets, to build the combined model, namely, the deep learning radiomic signature (DLR_Sig). We used the receiver operating characteristic (ROC) curve and the area under the ROC curve (AUC) to estimate the performance of the multiclass classification model.

**Results:**

The training set included 440 benign, 44 borderline, and 196 malignant ovarian tumours. The testing set included 109 benign, 11 borderline, and 49 malignant ovarian tumours. DLR_Sig three-class prediction model had the best overall and class-specific classification performance, with micro- and macro-average AUC of 0.90 and 0.84, respectively, on the testing set. Categories of identification AUC were 0.84, 0.85, and 0.83 for benign, borderline, and malignant ovarian tumours, respectively. In the confusion matrix, the classifier models of Clinic_Sig and Rad_Sig could not recognise borderline ovarian tumours. However, the proportions of borderline and malignant ovarian tumours identified by DLR_Sig were the highest at 54.55% and 63.27%, respectively.

**Conclusions:**

The three-class prediction model of US-based DLR_Sig can discriminate between benign, borderline, and malignant ovarian tumours. Therefore, it may guide clinicians in determining the differential management of patients with ovarian tumours.

**Supplementary Information:**

The online version contains supplementary material available at 10.1186/s12880-024-01251-2.

## Introduction

Ovarian tumours are of various histological types, including benign, borderline, and malignant lesions [1–3]. Benign tumours have good prognosis and are treated conservatively and with regular follow-up observations [2, 4]. Epithelial hyperplasia and nuclear atypia are more prominent in borderline ovarian tumours (BOTs) than in benign ovarian tumours; however, BOTs have no stromal invasion, unlike ovarian malignancies [5]. BOTs have good prognosis, with a 10-year survival rate of > 95% for stages I, II, and III [6]. The primary treatment for BOTs is surgical intervention; however, more than one-third of BOTs cases occur in women aged under 40 years who may want to conceive in the future [1]. Therefore, prioritising fertility preservation in young women desiring to have children is crucial. Patients with malignant ovarian tumours should be referred to gynaecologic oncologists for further diagnosis and treatment, and depending on the stage of cancer, debulking surgery and chemotherapy may be considered [7]. Different types of ovarian tumours have distinct clinical and pathological characteristics, treatment strategies, and prognoses. The early detection and treatment of ovarian malignancies can improve patient outcomes [8]. Therefore, the preoperative identification of the nature of ovarian tumours is critical for patients and can guide physicians in developing individualised and precise management plans.

Ultrasonography, especially transvaginal, is considered the primary method for evaluating adnexal tumours [9, 10]. Currently, subjective assessment by ultrasound (US) experts is a relatively good method of distinguishing the nature of ovarian tumours. However, US specialists are few, and differences in subjective diagnoses among US physicians with different experience levels exist [11, 12]. Therefore, objectively and quantitatively analysing the various imaging features that may reveal the potential biological characteristics of tumours in a reproducible manner is necessary.

Radiomics is an emerging field of quantitative imaging that can significantly impact personalised medicine. They can mine quantitative features from medical images using high-throughput methods, which are then transformed into objective and structured data through complex algorithms and applied to clinical decision support systems to improve diagnosis, prognosis assessment, and prediction accuracy [13, 14]. Previous studies on computed tomography (CT)/magnetic resonance imaging (MRI)/US-based radiomics for differentiating benign and malignant ovarian tumours achieved satisfactory diagnostic results [15–18]. However, radiomic features are predefined, including morphology, intensity, texture, and wavelet features, which are superficial and low-order, and cannot represent the heterogeneity of the entire tumour [19, 20]. Therefore, to accurately classify ovarian tumours, studying their deeper- and higher-level features is necessary.

Deep learning (DL) is a branch of machine learning (ML) that allows computing models with multiple processing layers to learn data representations at numerous abstraction levels [21]. The convolutional neural network (CNN) is the most commonly used DL architecture type in medical image analysis [22]. Suggestions that CNN-extracted features can provide various high-order features of images and apply them to specific clinical outcomes exist [20]. Successful application of DL requires a large number of training sets. However, medical data sets are often limited in number. Many practical applications currently use CNNs pre-trained on ImageNet, known as transfer learning (TL), to replace DL [23, 24]. Research using deep transfer learning (DTL) to classify benign and malignant ovarian tumours has been successful [11, 12, 25]. However, BOTs were categorised as malignant ovarian tumours for statistical analysis. Combining DL classification networks with traditional hand-crafted radiomics frameworks is a new development [26, 27]. Few reports exist on US-based combined DL radiomics (DLR) models as multi-classification prediction models for classifying ovarian tumours as benign, borderline, or malignant. We hypothesised that DLR could differentiate between benign, borderline, and malignant ovarian tumours. Hence, this study aimed to develop an US-based DLR to identify benign, borderline, and malignant ovarian lesions.

## Materials and methods

### Study design and participants

**Fig. 1:**
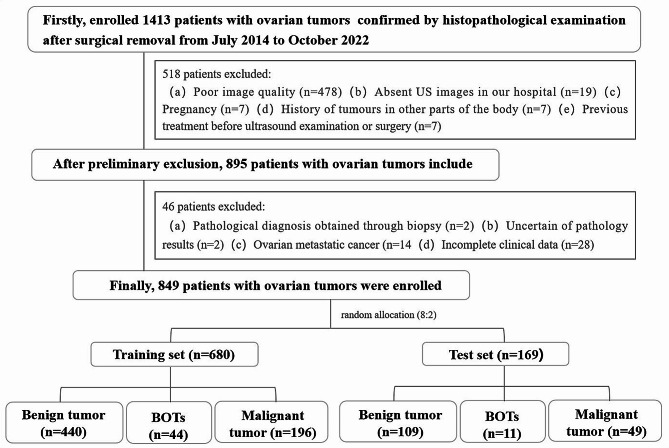
Inclusion and exclusion criteria for patients with ovarian tumours for the training and testing sets. Abbreviation: BOTs = borderline ovarian tumours

We enrolled 849 patients with ovarian tumours confirmed by histopathological examination after surgical removal from July 2014 to October 2022. The inclusion criteria were: (a) complete US examination within 1 month before surgery and (b) a clear and definite US image of the target lesion. The exclusion criteria were: (a) poor image quality, (b) absent or incomplete US and clinical data, (c) pregnancy, (d) history of tumours in other parts of the body and ovarian metastatic cancer, (e) previous treatment before US examination or surgery, and (f) pathological diagnosis obtained through biopsy and uncertain pathology results. A flowchart of the participants is shown in Fig. 1.

The study population was categorised into different labels based on pathological results, with benign ovarian tumours labelled as “class 0”, BOT as “class 1”, and malignant ovarian tumours as “class 2”. Participant data was randomised into training and testing sets in a ratio of 8:2 using Python’s statistical package. Our data random partitioning adopted a stratified method to handle imbalanced data between the training and testing sets; hence, the proportion of patients with benign, borderline, and malignant ovarian tumours in the total study population, training set, and testing set was similar. No data overlap occurred between the training and testing sets, avoiding the repeated use of data from the same patient [28].

The training set was used to learn the parameters and build the model, whereas the testing set was used to evaluate the generalisability of the selected model and prevent overfitting.

### Collecting clinical parameters

Preoperative clinical data of all patients, including age, menopausal status, height, weight, body mass index (BMI), carbohydrate antigen 125 (CA125), red blood cell count (RBC), white blood cell count (WBC), neutrophil count (N), lymphocyte count (L), monocyte count (M), platelet count (PLT), and haemoglobin were obtained from the patient’s electronic medical records. BMI and some inflammation-related risk factors, such as the neutrophil-to-lymphocyte ratio (NLR), derived neutrophil-to-lymphocyte ratio (dNLR), platelet-to-lymphocyte ratio (PLR), lymphocyte-to-monocyte ratio (LMR), and systemic immune-inflammation index (SII), were calculated using the following simple formulas:

$$ BMI=\frac{weight\left(kg\right)}{{height}^{2}\left({m}^{2}\right)}$$

$$ NLR=\frac{N\left({10}^{9}\right)}{L\left({10}^{9}\right)}$$

$$ dNLR=\frac{N\left({10}^{9}\right)}{(WBC-N)\left({10}^{9}\right)}$$

$$ PLR=\frac{PLT\left({10}^{9}\right)}{L\left({10}^{9}\right)}$$

$$ LMR=\frac{L\left({10}^{9}\right)}{M\left({10}^{9}\right)}$$

$$ SII=\frac{N\left({10}^{9}\right)\times PLT\left({10}^{9}\right)}{L\left({10}^{9}\right)}$$

### Ultrasound data acquisition

All participants underwent transvaginal ultrasonography whenever possible. If a mass was too large to be fully displayed on transvaginal ultrasonography, it could be supplemented with a transabdominal US. Transrectal or transabdominal ultrasonography could be performed if a patient was unsuitable for transvaginal ultrasonography. The following US equipment was used in the study: GE Voluson E10, GE Voluson E8, GE Healthcare (GE Medical Systems, Zipf, Austria), and Mindray Resona R9 (Mindray Bio-Medical Electronics Co., Ltd., China), with RIC5-9-D, V11-3HU transvaginal US probes, and C1-5-D and SC6-1U abdominal US probes. Recorded US semantic features included: maximum diameter of the lesion (≤ 50, 50–100, and ≥ 100 mm), characteristics of the mass (cystic, cystic-solid mixed, solid), colour Doppler score (1, no blood flow signal; 2, low blood flow signal; 3, moderate blood flow signal; 4, rich blood flow signal), laterality of the mass (unilateral or bilateral), and ascites (present or absent). If a patient had more than one ovarian mass, we selected the mass with the most complex morphology or the largest for further assessment [12, 29, 30].

### The specialized assessment of ultrasound images

Initially, the ultrasound image was assessed by Doctor A, a seasoned gynecology and obstetrics ultrasound specialist with ten years of professional experience, who provided the initial diagnosis. Subsequently, Doctor B, another gynecology and obstetrics ultrasound expert with over 15 years of experience, confirmed the diagnosis. In cases of discordant opinions, a senior expert in gynecology and obstetrics ultrasound with more than two decades of experience was consulted, leading to a consensus through collaborative discussion. These doctors were unaware of the patient’s clinical and biochemical indicators or pathological results.

### Image pre-processing and regions of interest (ROI) segmentation

The grey-level ranges of two-dimensional images obtained using different US devices vary significantly, and the voxel spacing of images obtained using different US devices are typically different. To address these problems, we employed a fixed-resolution resampling method.

The US images were imported into the ITK-SNAP 3.8.0 software (http://www.itksnap.org) for manual ROI segmentation. Segmentation of all ROI was completed by A (an US expert with > 10 years of experience) and confirmed by B (an US expert with > 15 years of experience). When there were differences in opinion, a senior physician (an US expert with > 20 years of experience) was consulted for joint decision-making. To ensure the robustness and repeatability of the extracted radiomics features, we randomly selected 50 US images from the dataset two weeks later, in which A re-delineated the ROIs and C (an US expert with 12 years of experience) independently delineated the ROI simultaneously. All the US experts were blinded to the clinical and pathological results of the study population.

For DTL, the slice of the US image with the largest tumour area was trimmed to represent each patient. The grey values were normalised to the range [-1, 1] using a min-max transformation. Then, each cropped subregion US image was resized to 224 × 224 by the nearest interpolation method and saved as a “.png” file to meet the requirements for input into a CNN model.

### Hand-crafted radiomics feature extraction and selection

We employed PyRadiomics (http://pyradiomics.readthedocs.io) to extract the handcrafted radiomic features. Subsequently, Z-score normalisation was performed to eliminate differences in the value scales of the extracted features.

**Fig. 2:**
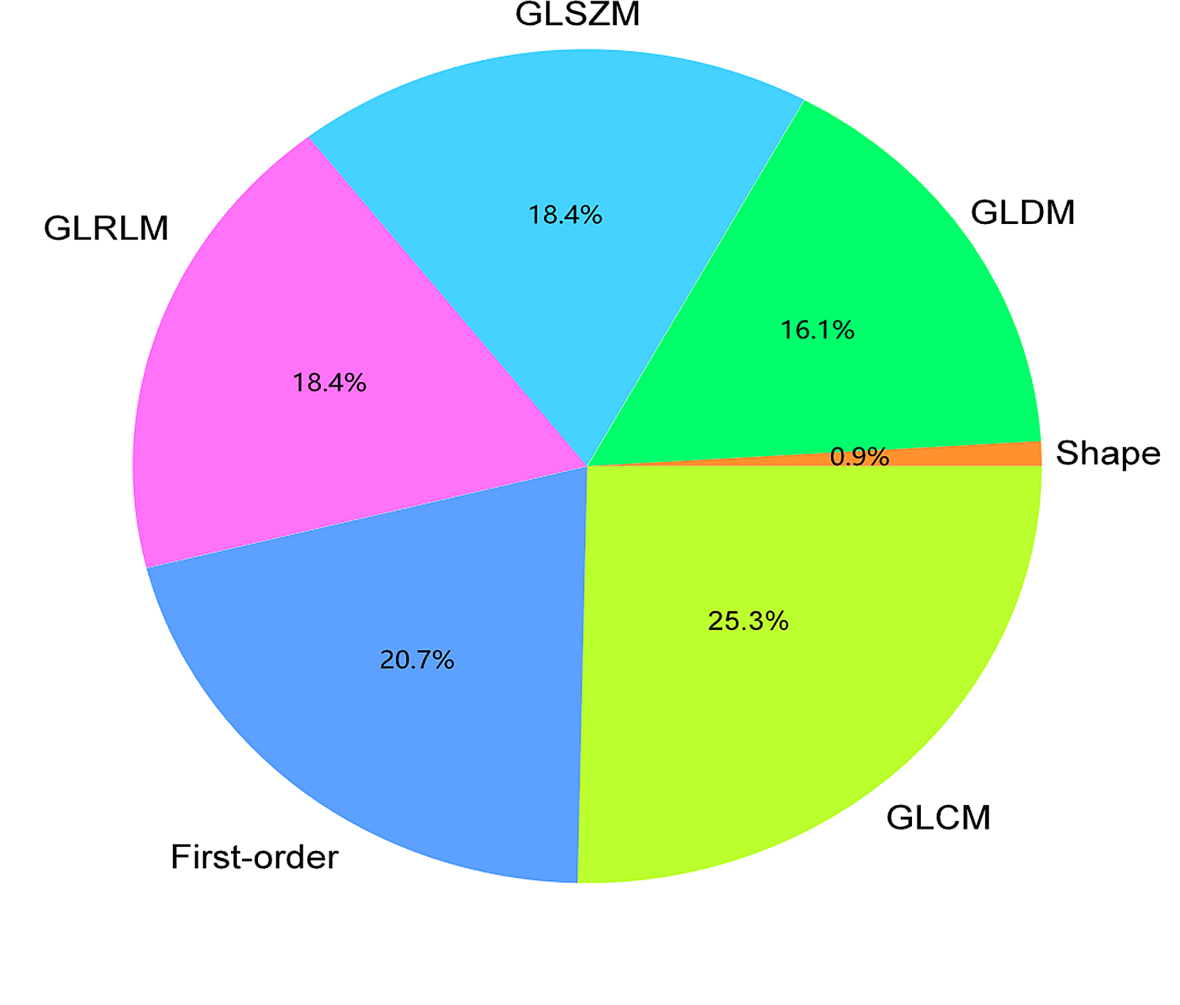
The proportion of hand-crafted radiomics features. Abbreviation: GLDM = grey-level dependence matrices, GLSZM = grey-level size zone matrices, GLRLM = grey-level run length matrices, GLCM = grey-level co-occurrence matrices

**Fig. 3:**
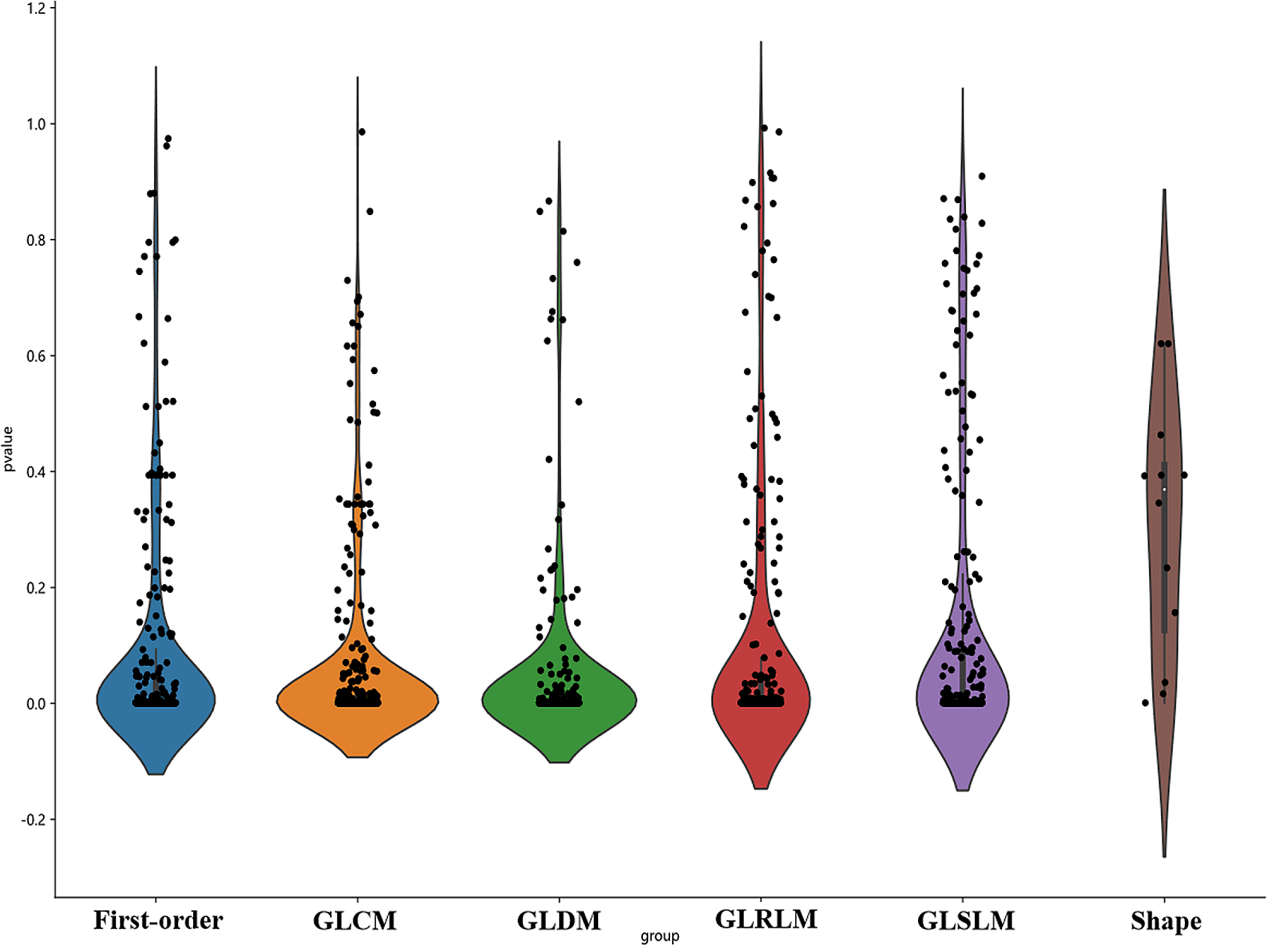
All hand-crafted radiomics features’ corresponding ***P***-value results. Abbreviation: GLDM = grey-level dependence matrices, GLSZM = grey-level size zone matrices, GLRLM = grey-level run length matrices, GLCM = grey-level co-occurrence matrices

A total of 1476 handcrafted radiomics features were extracted from tasks 1 and 2, including the first-order features, shape features, gray-level dependence matrix (GLDM), gray-level size zone matrix (GLSZM), gray-level run length matrix (GLRLM), and gray-level co-occurrence matrix (GLCM). The number and proportion of handcrafted radiomics features are presented in Fig. 2. The ***P***-values for all handcrafted features are shown in Fig. 3.

First, we retained hand-crafted radiomic features with intra-/inter-class correlation coefficient > 0.8, to ensure the robustness and repeatability of these features. Only 1,444 features with *P* < 0.05 after a T- or Mann–Whitney U-test were retained. Subsequently, spearman correlation analysis was used to calculate the correlation between features. A feature with a correlation coefficient of more than 0.9 between any two features is retained; thus, using a greedy recursive deletion strategy to maintain the features strongly correlated with the predicted target, 295 features were retained. Finally, least absolute shrinkage and selection operator (LASSO) regression algorithms were used for feature selection. Depending on the regulation weight λ, LASSO shrinks all regression coefficients towards zero and sets the coefficients of the irrelevant features precisely to zero. We employed 10-fold cross-validation with the minimum criteria to determine the optimal λ, where the final value of λ (0.016768) yielded the minimum cross-validation error. We retained 53 non-zero-coefficient features as optimal features.

### The deep transfer learning procedure

We used DTL, a CNN model pre-trained on the ImageNet dataset, to avoid overfitting owing to the limited size of the training dataset.

Data augmentation is often required to improve DTL’s prediction performance and generalisation ability in image classification because of imbalanced or insufficient data. Hence, we utilised horizontal flipping and random cropping for data augmentation, which helped increase the sample size and enhance the model performance.

To better perform the generalisation, we carefully set the learning rate. In this study, we adopted a cosine-decay learning rate algorithm. The learning rates are presented in Additional file 1.

### Signature building

The baseline clinical data were analysed in the training set. Clinical parameters and ultrasonic semantic features with *P* < 0.05 were selected, and spearman correlation analysis was used to determine the linear relationship between these parameters. Parameters without a significant linear correlation were inputted into the support vector machine model to build clinical signature (Clinic_Sig).

After the LASSO regression feature screening, the optimal features were input into the Light Gradient Boosting Machine (LightGBM) model to construct radiomic signature (Rad_Sig).

After the US image of the mass with the largest section was inputted into a ResNet50 model, the prediction probability of each sample was used as deep transfer learning (DTL_Sig). Gradient-weighted class activation mapping (Grad-CAM) was applied to visualise the internal network algorithm and explain the decision basis of the CNN model.

We fused the prediction results of Rad_Sig, DTL_Sig, and Clinic_Sig for each sample as new features, put them into the Gradient Boosting model, and constructed a combined model on the training set, namely deep learning radiomic signature (DLR_Sig).

### Model assessment

In this study, we employed a one-versus-rest method, which is often applied in multiclass classification. We evaluated the model’s performance based on receiver operating characteristic (ROC) curves and the area under the ROC curve (AUC). We used Precision, Recall, F1 score, macro-average, micro-average, and weighted average to assess the class of discrimination of one-versus-rest for the ovarian tumours of each group and the whole. A confusion matrix was used to analyse the errors in the model.

### Statistical analysis

Statistical analysis was performed using Python (https://www.python.org/). Normally distributed variables are reported as mean ± standard deviation, whereas non-normally distributed variables are reported as median (interquartile range). Categorical variables are expressed as frequencies (percentages). One-way analysis of variance was used to compare the three data groups with normality and homogeneity criteria, and a rank-sum nonparametric test for multiple independent samples was adopted for variables with no normality and homogeneity. Categorical data were analysed using the chi-square (χ^2^) test. A two-sided *P*<0.05 was considered statistically significant.

## Results

### Patient characteristics

We included 849 patients in this study. Among them, 549 (64.66%), 55 (6.48%), and 245 (28.86%) had benign, borderline, and malignant ovarian tumours, respectively. The proportions of benign, borderline, and malignant ovarian tumours in the entire study group, training set, and testing set were approximately the same. The baseline characteristics are shown in Table 1.

**Table 1:** Training and testing sets of clinical parameters and semantic features of ultrasound

	Training set (*n* = 680)					Testing set (*n* = 169)			
	Benign tumour(*n* = 440)	BOTs(*n* = 44)	Malignant tumour(*n* = 196)	P		Benign tumour(*n* = 109)	BOTs(*n* = 11)	Malignant tumour(*n* = 49)	P
age (y)	37.07 ± 12.38	42.16 ± 13.71	49.63 ± 13.59	< 0.001*		38.70 ± 14.13	39.64 ± 14.66	48.47 ± 10.41	< 0.001*
Height (m)	1.58 ± 0.06	1.58 ± 0.058	1.56 ± 0.06	< 0.001*		1.57 ± 0.06	1.58 ± 0.03	1.56 ± 0.04	0.110
Weight (kg)	54.67 ± 9.54	56.51 ± 8.49	55.16 ± 9.01	0.468		55.85 ± 9.25	55.11 ± 11.13	54.68 ± 9.52	0.467
BMI	21.95 ± 3.56	22.68 ± 3.25	22.65 ± 3.29	0.014*		22.53 ± 3.58	22.12 ± 3.97	22.46 ± 3.73	0.882
RBC_count (10^12^/L)	4.55 ± 0.53	4.46 ± 0.41	4.36 ± 0.57	< 0.001*		4.31 ± 0.55	4.41 ± 0.55	4.30 ± 0.54	0.975
WBC_count (10^9^/L)	7.03 ± 2.00	6.80 ± 2.20	7.79 ± 2.58	< 0.001*		7.26 ± 2.77	6.20 ± 0.94	7.11 ± 1.74	0.623
Neutrophil_count (10^9^/L)	4.18 ± 1.788	4.31 ± 2.16	5.22 ± 2.44	< 0.001*		4.52 ± 2.62	3.18 ± 0.89	4.72 ± 1.89	0.780
Lymphocyte_count (10^9^/L)	2.12 ± 0.62	1.87 ± 0.63	1.78 ± 0.74	< 0.001*		2.06 ± 0.70	2.32 ± 0.74	2.03 ± 1.15	0.906
Monocyte_count (10^9^/L)	0.63 ± 0.28	0.55 ± 0.25	0.64 ± 0.28	0.852		0.49 ± 0.19	0.52 ± 0.15	0.51 ± 0.23	0.564
PLT_count (10^9^/L)	296.91 ± 72.08	275.25 ± 56.68	345.37 ± 115.83	< 0.001*		282.19 ± 65.22	275.09 ± 82.76	340.04 ± 89.81	< 0.001*
Hemoglobin(g/L)	124.44 ± 18.09	123.34 ± 14.20	117.22 ± 16.54	< 0.001*		119.75 ± 16.99	117.18 ± 21.75	114.90 ± 16.21	0.097
NLR	2.20 ± 1.59	2.91 ± 2.73	3.51 ± 2.43	< 0.001*		2.72 ± 3.17	1.56 ± 0.75	2.78 ± 1.91	0.991
PLR	154.04 ± 83.09	168.43 ± 94.34	228.71 ± 137.61	< 0.001*		151.78 ± 64.23	130.95 ± 53.74	200.41 ± 118.78	0.002*
LMR	3.82 ± 1.62	3.87 ± 1.63	3.40 ± 2.60	0.016*		4.51 ± 1.69	4.72 ± 1.96	4.29 ± 1.76	0.510
dNLR	1.57 ± 1.14	1.76 ± 2.50	5.61 ± 47.72	0.073		1.88 ± 1.66	1.15 ± 0.51	1.99 ± 1.53	0.799
SII	663.44 ± 534.53	807.60 ± 843.56	1258.88 ± 1026.20	< 0.001*		752.95 ± 916.38	430.17 ± 211.21	969.97 ± 779.50	0.190
Menopausal_state, n (%)				< 0.001*					< 0.001*
Premenopausal	383(87.05%)	28(63.64%)	102(52.04%)			90(82.57%)	9(81.82%)	25(51.02%)	
Menopause	57(12.95%)	16(36.36%)	94(47.96%)			19(17.43%)	2(18.18%)	24(48.98%)	
Tumour_diameter (mm), n (%)				< 0.001*					0.001*
≤ 50	68(15.45%)	6(13.64%)	12(6.12%)			21(19.27%)	1(9.09%)	1(2.04%)	
50–100	293(66.59%)	18(40.91%)	79(40.31%)			59(54.13%)	5(45.45%)	20(40.82%)	
≥ 100	79(17.95%)	20(45.45%)	105(53.57%)			29(26.61%)	5(45.45%)	28(57.14%)	
Mass_characteristicn, n (%)				< 0.001*					< 0.001*
Cystic	314(71.36%)	18(40.91%)	13(6.63%)			79(72.48%)	4(36.36%)	1(2.04%)	
Cystic-solid mixed	102(23.18%)	21(47.73%)	89(45.41%)			23(21.10%)	6(54.55%)	30(61.22%)	
Solid	24(5.45%)	5(11.36%)	94(47.96%)			7(6.42%)	1(9.09%)	18(36.73%)	
colour_score, n (%)				< 0.001*					< 0.001*
1	401(91.14%)	8(18.18%)	11(5.61%)			83(76.15%)	4(36.36%)	2(4.08%)	
2	35(7.95%)	24(54.55%)	77(39.29%)			21(19.27%)	7(63.64%)	22(44.90%)	
3	4(0.91%)	10(22.73%)	91(46.43%)			4(3.67%)	0	25(51.02%)	
4	0	2(4.55%)	17(8.67%)			1(0.92%)	0	0	
Ascites, n (%)				< 0.001*					< 0.001*
No	437(99.32%)	39(88.64%)	138(70.41%)			107(98.17%)	11(100.00%)	40(81.63%)	
Yes	3(0.68%)	5(11.36%)	58(29.59%)			2(1.83%)	0	9(18.37%)	
Tumour_side, n (%)				< 0.001*					0.072
Unilateral	379(86.14%)	42(95.45%)	124(63.27%)			99(90.83%)	9(81.82%)	38(77.55%)	
Bilateral	61(13.86%)	2(4.55%)	72(36.73%)			10(9.17%)	2(18.18%)	11(22.45%)	
CA125_level (U/ml), n (%)				< 0.001*					< 0.001*
≤ 35	304(69.09%)	27(61.36%)	46(23.47%)			80(73.39%)	6(54.55%)	14(28.57%)	
>35, ≤ 200	115(26.14%)	11(25.00%)	51(26.02%)			26(23.85%)	4(36.36%)	13(26.53%)	
>200, <500	16(3.64%)	2(4.55%)	34(17.35%)			2(1.83%)	0	8(16.33%)	
≥ 500	5(1.14%)	4(9.09%)	65(33.16%)			1(0.92%)	1(9.09%)	14(28.57%)	

BOTs: borderline ovarian tumours; BMI: body mass index; RBC: red blood cell; WBC: white blood cell; NLR: neutrophil-to-lymphocyte ratios; PLR: platelet-to-lymphocyte ratios; LMR: lymphocyte-to-monocyte ratios; dNLR: derived neutrophil-to-lymphocyte ratios; SII: systemic immune-inflammation index; CA125: carbohydrate antigen 125; *: *P*<0.05

### The ultrasound expert assessment the benign, borderline, and malignant ovarian tumours

Ultrasound specialists demonstrated a high level of accuracy in distinguishing between benign and malignant ovarian tumours, with rates of 95.80% and 82.80%, respectively. Conversely, the accuracy in identifying borderline ovarian tumours was notably lower at 34.50% (Table 2).

**Table 2:** The expert assessment the ultrasound images

		Histopathological diagnosis			Sum
		Benign tumour (n, Ac%)	BOT (n, Ac%)	Malignant tumour (n, Ac%)	
US expert examiners	Benign tumour (n, Ac%)	505 (95.80%)	10	12	527
	BOTs (n, Ac%)	24	19 (34.50%)	12	55
	Malignant tumour (n, Ac%)	20	26	221 (82.80%)	267
Sum		549	55	245	849

US, ultrasound; Ac, Accuracy; BOT, borderline ovarian tumour

### The confusion matrix of the three-class classification prediction model

**Fig. 4:**
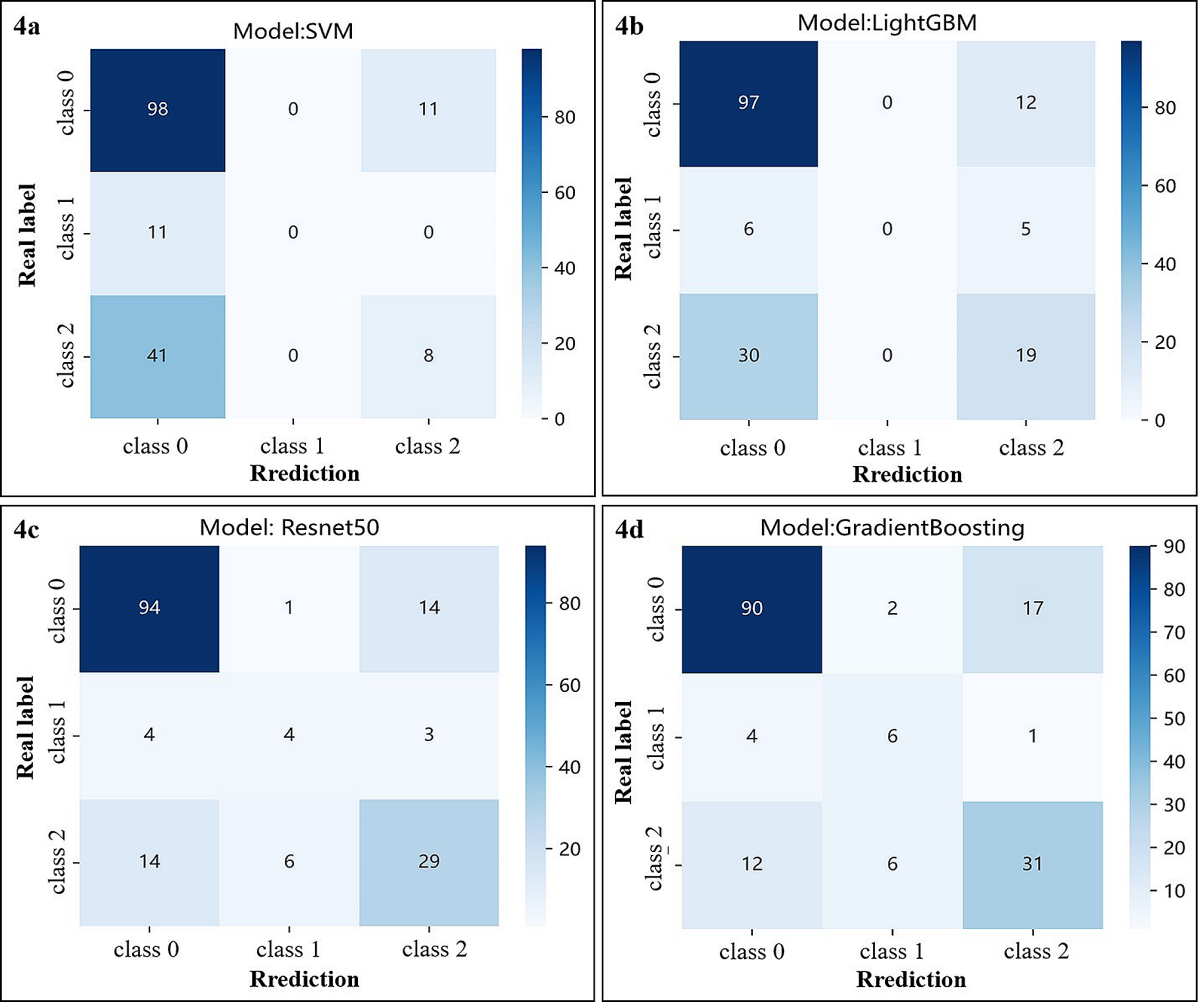
Confusion matrix of three-class classification results based on the test set. (**4a**) Clinic_Sig; (**4b**) Rad_Sig; (**4c**) DTL_Sig; (**4d**) DLR_Sig. Class 0: benign ovarian tumours; class 1: BOT; class 2: malignant ovarian tumours. LightGBM, Light Gradient Boosting Machine

We used the confusion matrix to understand where the classifier model made the classification errors and their proportions (Fig. 4; Table 3). These multiclass classification prediction models had a high rate of correctly distinguishing benign ovarian tumours, 89.91%, 88.99%, 86.24%, and 82.57%, respectively). Clinic_Sig and Rad_Sig showed relatively poor accuracy in determining malignant ovarian tumours (16.33% and 38.78%, respectively). The classifier models Clinic_Sig and Rad_Sig cannot recognise BOT. The proportion of BOT identified by DLR was the highest at 54.55%.

**Table 3:** The error analysis of the three-class classification prediction model

Signature	Real label	class	Prediction			sum
			Benign ovarian tumour	BOT	Malignant ovarian tumour	
Clinic_Sig	Real label	Benign ovarian tumour	98(89.91%)	0(0%)	11(10.09%)	109
		BOT	11(100%)	0(0%)	0(0%)	11
		Malignant ovarian tumour	41(83.67%)	0(0%)	8(16.33%)	49
		sum	150	0	19	169
Rad_Sig	Real label	Benign ovarian tumour	97(88.99%)	0(0%)	12(11.01%)	109
		BOT	6(54.55%)	0(0%)	5(45.45%)	11
		Malignant ovarian tumour	30(61.22%)	0(0%)	19(38.78%)	49
		sum	133	0	36	169
DTL_Sig	Real label	Benign ovarian tumour	94(86.24%)	1(0.92%)	14(12.84%)	109
		BOT	4(36.36%)	4(36.36%)	3(27.28%)	11
		Malignant ovarian tumour	14(28.58%)	6(12.24%)	29(59.18%)	49
		sum	112	11	46	169
DRL_Sig	Real label	Benign ovarian tumour	90(82.57%)	2(1.83%)	17(15.60%)	109
		BOT	4(36.36%)	6(54.55%)	1(9.09%)	11
		Malignant ovarian tumour	12(24.49%)	6(12.24%)	31(63.27%)	49
		sum	106	14	49	169

Clinic_Sig: clinical signature; Rad_Sig: radiomics signature; DTL_Sig: deep transfer learning signature; DLR_Sig: deep learning radiomic signature; BOT: Borderline ovarian tumour

### Classification performance

**Fig. 5:**
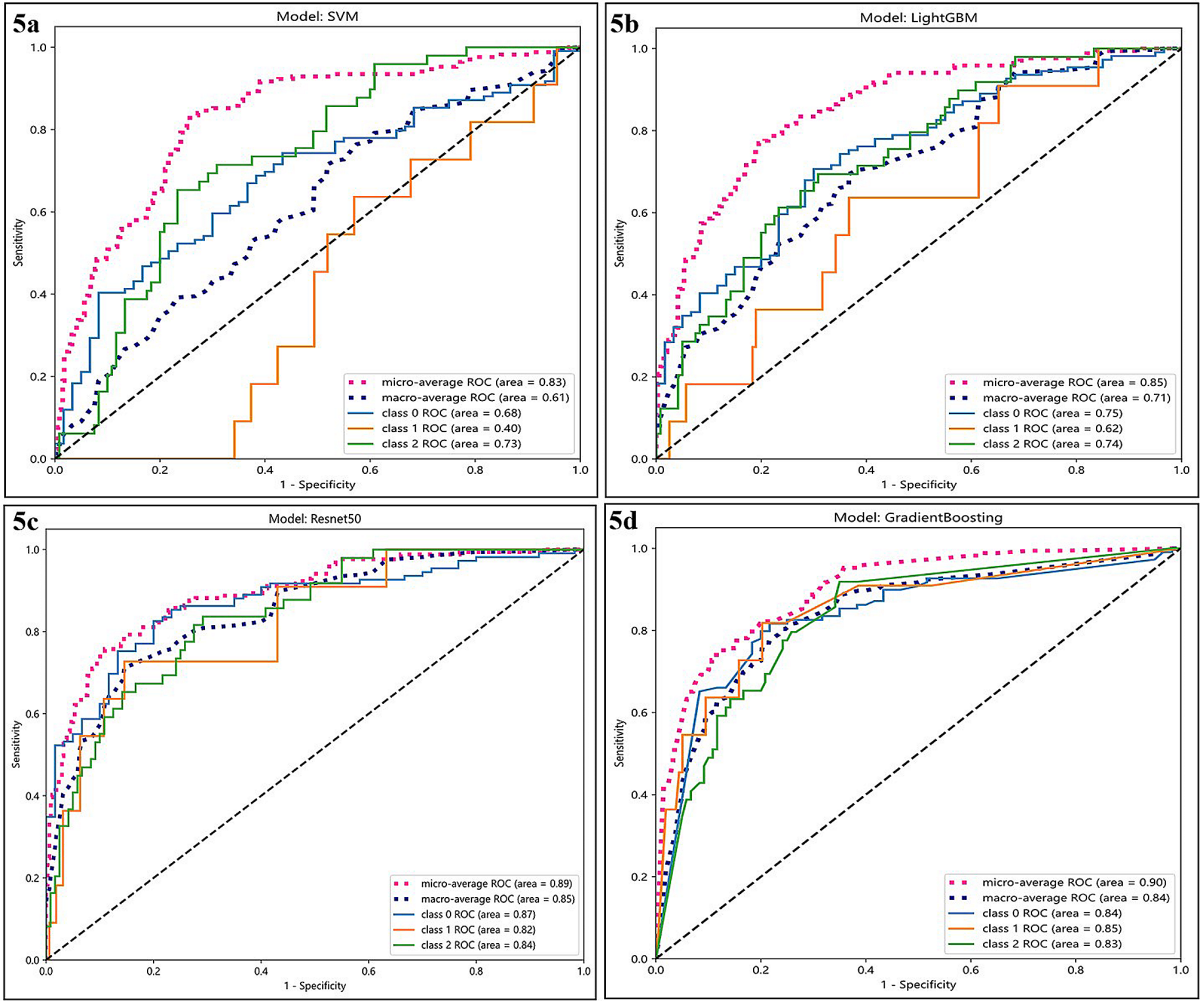
Three-class (one-vs-rest) ROC of the test set. (**5a**) Clinic_Sig; (**5b**) Rad_Sig; (**5c**) DTL_Sig; (**5d**) DLR_Sig. Class 0: benign ovarian tumours; class 1: BOT; class 2: malignant ovarian tumours. Micro- and macro-average ROC indicated the overall distinguishing ability of the three-class classification. LightGBM, Light Gradient Boosting Machine

The DLR_Sig three-class prediction model had the best overall and class-specific classification performance, with the micro/macro average AUC 0.90 and 0.84 on the testing set, respectively. The categories of identification AUC were 0.84 for benign, 0.85 for borderline, and 0.83 for malignant ovarian tumours (Fig. 5; Table 4).

**Table 4:** Overall and class-specific classification performance

		AUC	Precision (%)	Recall (%)	F1 Score (%)	Acc (%)
Clinic_Sig	Benign ovarian tumour	0.68	65.33	89.91	75.68	62.72
	BOT	0.40	0.00	0.00	0.00	93.49
	Malignant ovarian tumour	0.73	42.11	16.33	25.40	69.23
	micro-average	0.83	62.72	62.72	62.72	62.72
	macro-average	0.61	35.81	35.41	33.69	75.15
	weighted-average		54.35	62.73	48.81	66.61
Rad_Sig	Benign ovarian tumour	0.75	72.93	88.99	80.17	71.60
	BOT	0.62	0.00	0.00	0.00	93.49
	Malignant ovarian tumour	0.74	52.78	38.78	44.71	72.19
	micro-average	0.85	68.64	68.64	68.64	68.64
	macro-average	0.71	41.90	42.59	41.63	79.09
	weighted-average		62.341	68.64	64.67	73.20
DTL_Sig	Benign ovarian tumour	0.87	83.93	86.24	85.07	62.72
	BOT	0.82	36.36	36.36	36.36	91.72
	Malignant ovarian tumour	0.84	63.04	59.18	61.05	78.11
	micro-average	0.89	75.15	75.14	75.14	75.14
	macro-average	0.85	61.11	60.59	60.83	77.52
	weighted-average		74.78	75.15	74.94	69.07
DLR_Sig	Benign ovarian tumour	0.84	84.91	82.57	83.72	79.29
	BOT	0.85	42.86	54.55	57.14	93.31
	Malignant ovarian tumour	0.83	63.27	63.27	63.27	78.70
	micro-average	0.90	75.14	75.14	75.14	75.14
	macro-average	0.84	63.68	66.80	68.04	83.77
	weighted-average		75.90	75.15	76.06	80.03

Clinic_Sig: clinical signature; Rad_Sig: radiomics signature; DTL_Sig: deep transfer learning signature; DLR_Sig: deep learning radiomic signature; Acc: Accuracy; BOT: Borderline ovarian tumour

### Application of grad-CAM

**Fig. 6:**
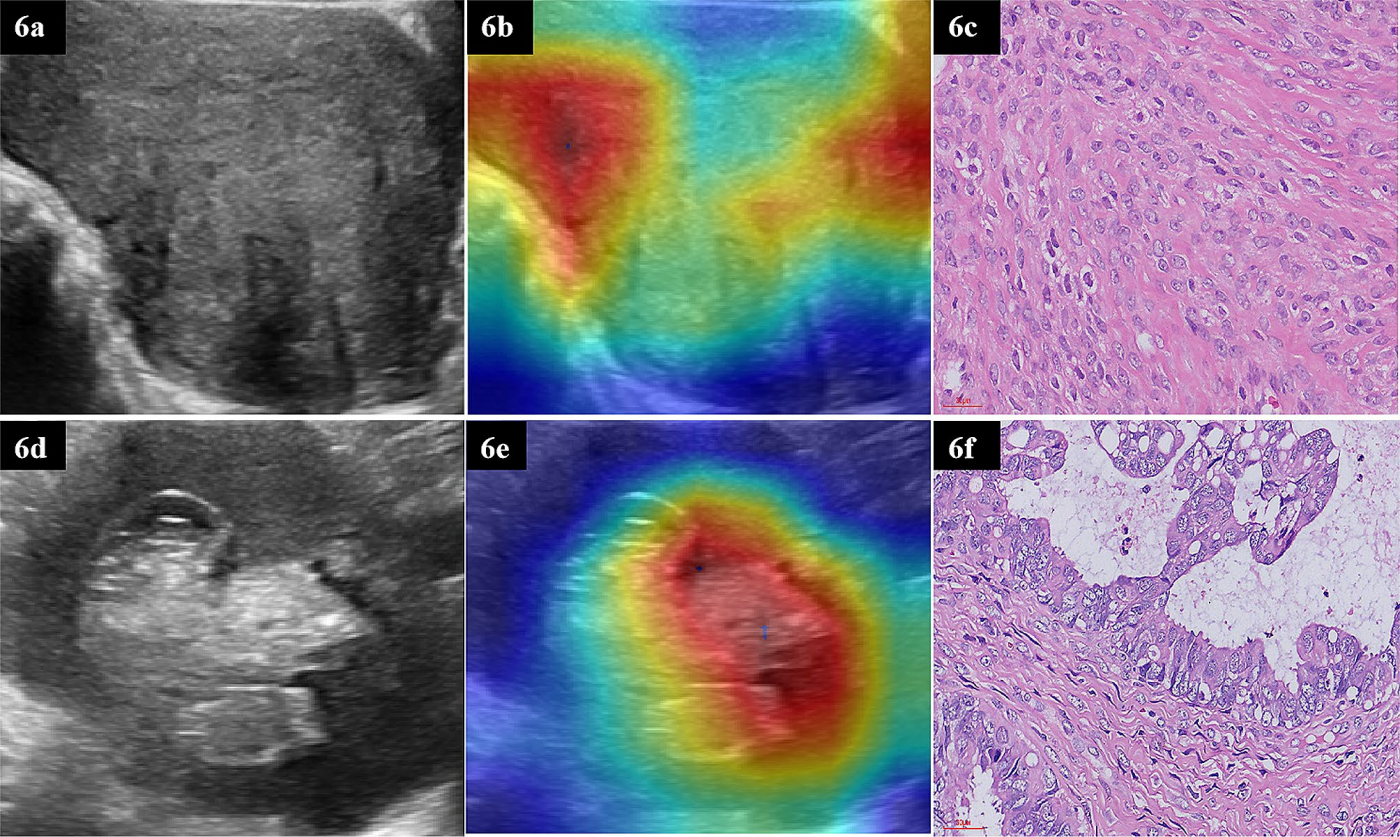
The Resnet50 model with Grad-CAM was used on ovarian tumour patients. (**6a–c**) A solid hypoechoic mass in one patient’s pelvis, 100 mm in diameter. (**6a**) US image; (**6b**) Grad-CAM; the red area is the basis of decision-making for Resnet50; (**6c**) Histopathological results: theca cell tumour (40x). (**6d–f**) A cystic-solid mixed mass in a patient’s pelvis, 112 mm in diameter. (**6d**) US image; (**6e**) Grad-CAM; the red area is the basis of decision-making for Resnet50;(**6f**) Histopathological results: borderline ovarian tumour (40x)

Grad-CAM, which can produce a coarse localisation map highlighting the critical regions for classification targets, is proposed as a method for visualising the decisions of CNN models. The red areas of the heat map are crucial references for model decision-making [31]. The site of concern for US diagnosis is consistent with the area of concern for CNN decision making (Fig. 6).

In Fig. 6a, b, and c, there was a solid low-echoic mass in one patient’s pelvis, 100 mm in diameter. Rich blood flow signals were observed in and around the mass, with a CA125 of 8.67 U/ml. An US expert suggested that the patient had a malignant ovarian tumour. However, DTL_Sig predicted benign lesions with a probability of 97.35%. Pathological results showed that it was a benign theca cell tumour. The prediction of DTL_Sig was highly consistent with the pathological diagnosis.

In Fig. 6d, e, and f, there was a cystic-solid mixed mass in a patient’s pelvis, which was 112 mm in diameterand had a CA125 level of 206 U/ml. An US expert suggested that the patient had a malignant ovarian tumour. However, DTL_Sig indicated a BOT with an 85.98% probability. A pathological diagnosis of BOT was made. DTL_Sig prediction was highly consistent with the pathological diagnosis.

## Discussion

The accurate prediction of the category of ovarian tumours is critical for patient-centred care. Studies on the multiclass classification of DLR to classify ovarian tumours are relatively scarce. In this study, we constructed four multiclassification prediction models to classify benign, borderline, and malignant ovarian tumours. We found that the DLR prediction model had the optimum ability to classify ovarian tumours and generalise the testing set.

Ultrasonography is the primary method for screening ovarian tumours. Serum tumour markers are essential for discovering and treating ovarian cancer, and CA125 is the most important biomarker for evaluating ovarian cancer [32]. Inflammation is vital in the development and progression of ovarian cancer [33]. Therefore, we collected US semantic features, serum tumour markers, and related inflammatory factors from the study population. These US semantic features and clinical parameters are typically obtained during routine examinations and do not add additional burden to the patient. We selected some semantic elements, serum tumour markers, and related inflammatory factors to construct Clinic_Sig. The Clinic_Sig three-class prediction model had poor overall and class-specific classification performance and could not predict BOT; the precision, recall, and F1 scores were all zero.

The US examinations were subjective. US experts have higher diagnostic accuracy than less experienced doctors; however, US experts are few [11]. Recently, radiomics has become a powerful new method for quantifying features from medical images, including potential pathophysiological information of reference cancer tissues [34]. Some studies have used MRI/CT/US-based radiomics to differentiate between benign and malignant ovarian tumours with higher diagnostic performance [15, 35, 36]. However, these studies did not mention the classification of BOT. Qi et al. [16] established and validated US-based radiomics models to discriminate between benign, borderline, and malignant serous ovarian tumours and provided preoperative diagnostic information to differentiate the nature of ovarian tumours. However, this was a binary classification study. In our research, the Rad_Sig three-class prediction model could not predict BOT, and the precision, recall, and F1 scores were all zero.

DL is becoming increasingly essential for image pattern recognition [21]. Considering the limited scale of medical datasets, we used TL to replace DL. TL is beneficial because it improves the performance of a model built on small samples by utilising the knowledge learned in similar classification tasks [28]. Gao et al. [25] and Christiansen et al. [11] developed a DTL model to identify benign and malignant ovarian tumours, equivalent to the diagnostic level of an US specialist. Chen et al. [12] developed DTL algorithms to distinguish malignant from benign ovarian tumours, comparable to expert subjective and ovarian adnexal reporting and data system assessments. However, they classified BOT as malignant ovarian tumours for statistical analysis. We used models pre-trained on ImageNet Resnet50 [11, 37]. The DTL_Sig three-class prediction model had good overall and class-specific classification performance, with the micro/macro average AUC 0.89 and 0.85 on the test set, respectively. Categories of identification AUC were 0.87 for benign, 0.82 for borderline, and 0.84 for malignant ovarian tumours. Although DTL performs well in various classification prediction tasks, it is a black-box algorithm that lacks interpretability, which restricts its application [31, 38]. Grad-CAM is employed as a method of depicting the decision-making of DL. In our study, as shown in Fig. 6, the site of concern for US experts making the diagnosis was consistent with the area of concern for CNN decision-making using Grad-CAM, and the DTL_Sig predictions were highly compatible with the pathological diagnosis results.

The combination of traditional manual radiomics and DTL algorithms, namely DLR, can effectively improve the accuracy and reliability of model predictions. It is currently a popular topic in ML for tumour research. Many studies [20, 38–40] show that the DLR model has a better prediction efficacy than Rad_Sig or DTL_Sig alone. The fusion process of data between traditional radiomics and DTL includes the fusion of features and decision levels, and the fusion of features often leads to overfitting because of many features [38]. We constructed a combined model for the training set by fusing the predicted probabilities of Clinic_Sig, Rad_Sig, and DTL_Sig for each sample. The combined three-class prediction model, DLR_Sig, had the best overall and class-specific classification performance, with the micro/macro average AUC 0.90 and 0.84 on the testing set, respectively. Categories of identification AUC were 0.84 for benign, 0.85 for borderline, and 0.83 for malignant ovarian tumours. The combined three-class prediction model performance for predicting BOT was the best, and the categories of identification AUC, Precision, Recall, F1 score, and accuracy had the highest performances of 0.85, 42.86%, 54.55%, 57.14%, and 93.31%, respectively. The prevalence of BOTs predicted by DLR_Sig (54.55%) exceeded that determined by ultrasound experts (34.50%).

This study had limitations. First, this was a retrospective single-centre study with a small sample size. Larger prospective and multicentre studies are required to evaluate the applicability of predictive models in clinical practice. Second, owing to the strict inclusion and exclusion criteria for data in this study, bias could have been introduced in the model’s training. Thirdly, in this study, we extracted features from two-dimensional US images. In future studies, we will include other modalities such as colour Doppler flow imaging, spectral Doppler imaging, and contrast-enhanced US to provide more predictive information. Lastly, ROI delineation and cropping of the top section of the tumour represented only one slice of the lesion and could not describe the heterogeneity of the entire tumour. In the future, we plan to store dynamic images of the whole tumour and input them into ML to obtain more comprehensive information.

## Conclusion

We developed a combined multiclass classification model that integrated clinical and traditional radiomics with DTL decision-level information to discriminate the nature of ovarian tumours. The performance and generalisation of this model have intensified its feasibility for distinguishing between benign, borderline, and malignant ovarian tumours.

### Electronic supplementary material

Below is the link to the electronic supplementary material.

Supplementary Material 1

## Data Availability

The datasets used and analyzed during the current study are available from the corresponding author upon reasonable request.

## Abbreviations

US: ultrasound
ML: machine learning
CNN: convolutional neural network
Clinic_Sig: clinical signatures
Rad_Sig: radiomic signature
DTL_Sig: deep transfer learning signature
DLR_Sig: deep learning radiomic signature
AUC: area under the curve of ROC
BOTs: borderline ovarian tumours
CT: computed tomography
MRI: magnetic resonance imaging
DL: deep learning
TL: transfer learning
DTL: deep transfer learning
DLR: DL radiomics
BMI: body mass index
CA125: carbohydrate antigen 125
RBC: red blood cell
WBC: white blood cell
N: neutrophil
L: lymphocyte
M: monocyte
PLT: platelet count
NLR: neutrophil-to-lymphocyte ratio
dNLR: derived neutrophil-tolymphocyte ratio
PLR: platelet-to-lymphocyte ratio
LMR: lymphocyte-to- monocyte ratio
SII: systemic immune-inflammation index
ROI: regions of interest
GLDM: grey-level dependence matrices
GLSZM: grey-level size zone matrices
GLRLM: grey-level run length matrices
GLCM: grey-level co-occurrence matrices
LASSO: least absolute shrinkage and selection operator
LightGBM: light Gradient Boosting Machine
Grad-CAM: Gradient-weighted class activation mapping
ROC: receiver operating characteristic curves

## References

[1] Maramai M, Barra F, Menada MV, Stigliani S, Moioli M, Costantini S (2020). Borderline ovarian tumours: management in the era of fertility-sparing surgery. Ecancermedicalscience.

[2] Sayasneh A, Ekechi C, Ferrara L, Kaijser J, Stalder C, Sur S (2015). The characteristic ultrasound features of specific types of ovarian pathology (review). Int J Oncol.

[3] Jayson GC, Kohn EC, Kitchener HC, Ledermann JA (2014). Ovarian cancer. Lancet (London England).

[4] Meys EMJ, Jeelof LS, Achten NMJ, Slangen BFM, Lambrechts S, Kruitwagen R (2017). Estimating risk of malignancy in adnexal masses: external validation of the ADNEX model and comparison with other frequently used ultrasound methods. Ultrasound Obstet Gynecology: Official J Int Soc Ultrasound Obstet Gynecol.

[5] Prat PJ (2017). Pathology of borderline and invasive cancers. Best Pract Res Clin Obstet Gynecol.

[6] May J, Skorupskaite K, Congiu M, Ghaoui N, Walker GA, Fegan S (2018). Borderline Ovarian tumors: Fifteen Years’ experience at a Scottish Tertiary Cancer Center. Int J Gynecol cancer: Official J Int Gynecol Cancer Soc.

[7] Fung-Kee-Fung M, Kennedy EB, Biagi J, Colgan T, D’Souza D, Elit LM (2015). The optimal organization of gynecologic oncology services: a systematic review. Curr Oncol (Toronto Ont).

[8] Reid BM, Permuth JB, Sellers TA (2017). Epidemiology of ovarian cancer: a review. Cancer Biology Med.

[9] Borrelli GM, de Mattos LA, Andres MP, Gonçalves MO, Kho RM, Abrão MS (2017). Role of imaging tools for the diagnosis of Borderline ovarian tumors: a systematic review and Meta-analysis. J Minim Invasive Gynecol.

[10] Chen H, Qian L, Jiang M, Du Q, Yuan F, Feng W (2019). Performance of IOTA ADNEX model in evaluating adnexal masses in a gynecological oncology center in China. Ultrasound Obstet Gynecology: Official J Int Soc Ultrasound Obstet Gynecol.

[11] Christiansen F, Epstein EL, Smedberg E, Åkerlund M, Smith K, Epstein E (2021). Ultrasound image analysis using deep neural networks for discriminating between benign and malignant ovarian tumors: comparison with expert subjective assessment. Ultrasound Obstet Gynecology: Official J Int Soc Ultrasound Obstet Gynecol.

[12] Chen H, Yang BW, Qian L, Meng YS, Bai XH, Hong XW (2022). Deep learning prediction of ovarian malignancy at US compared with O-RADS and Expert Assessment. Radiology.

[13] Lambin P, Leijenaar RTH, Deist TM, Peerlings J, de Jong EEC, van Timmeren J (2017). Radiomics: the bridge between medical imaging and personalized medicine. Nat Reviews Clin Oncol.

[14] Yip SS, Aerts HJ (2016). Applications and limitations of radiomics. Phys Med Biol.

[15] Zhang H, Mao Y, Chen X, Wu G, Liu X, Zhang P (2019). Magnetic resonance imaging radiomics in categorizing ovarian masses and predicting clinical outcome: a preliminary study. Eur Radiol.

[16] Qi L, Chen D, Li C, Li J, Wang J, Zhang C (2021). Diagnosis of ovarian neoplasms using Nomogram in Combination with Ultrasound Image-based Radiomics signature and clinical factors. Front Genet.

[17] Song XL, Ren JL, Zhao D, Wang L, Ren H, Niu J (2021). Radiomics derived from dynamic contrast-enhanced MRI pharmacokinetic protocol features: the value of precision diagnosis ovarian neoplasms. Eur Radiol.

[18] Yu XP, Wang L, Yu HY, Zou YW, Wang C, Jiao JW (2021). MDCT-Based Radiomics features for the differentiation of Serous Borderline ovarian tumors and serous malignant ovarian tumors. Cancer Manage Res.

[19] Gao W, Wang W, Song D, Yang C, Zhu K, Zeng M (2022). A predictive model integrating deep and radiomics features based on gadobenate dimeglumine-enhanced MRI for postoperative early recurrence of hepatocellular carcinoma. Radiol Med.

[20] Liu P, Liang X, Liao S, Lu Z (2022). Pattern classification for ovarian tumors by Integration of Radiomics and Deep Learning features. Curr Med Imaging.

[21] LeCun Y, Bengio Y, Hinton G (2015). Deep learning. Nature.

[22] Chartrand G, Cheng PM, Vorontsov E, Drozdzal M, Turcotte S, Pal CJ (2017). Deep learning: a primer for radiologists. Radiographics: Rev Publication Radiological Soc North Am Inc.

[23] Bo L, Zhang Z, Jiang Z, Yang C, Huang P, Chen T (2021). Differentiation of Brain Abscess from cystic glioma using conventional MRI based on deep transfer learning features and hand-crafted Radiomics features. Front Med.

[24] Feng B, Huang L, Liu Y, Chen Y, Zhou H, Yu T (2021). A transfer learning Radiomics Nomogram for Preoperative Prediction of Borrmann Type IV gastric Cancer from primary gastric lymphoma. Front Oncol.

[25] Gao Y, Zeng S, Xu X, Li H, Yao S, Song K (2022). Deep learning-enabled pelvic ultrasound images for accurate diagnosis of ovarian cancer in China: a retrospective, multicentre, diagnostic study. Lancet Digit Health.

[26] Han W, Qin L, Bay C, Chen X, Yu KH, Miskin N (2020). Deep transfer learning and Radiomics Feature Prediction of Survival of patients with high-Grade Gliomas. AJNR Am J Neuroradiol.

[27] Hu X, Zhou J, Li Y, Wang Y, Guo J, Sack I (2022). Added value of viscoelasticity for MRI-Based prediction of Ki-67 expression of Hepatocellular Carcinoma using a deep learning combined Radiomics (DLCR) Model. Cancers.

[28] Zhang Y, Hong D, McClement D, Oladosu O, Pridham G, Slaney G (2021). Grad-CAM helps interpret the deep learning models trained to classify multiple sclerosis types using clinical brain magnetic resonance imaging. J Neurosci Methods.

[29] Cao L, Wei M, Liu Y, Fu J, Zhang H, Huang J (2021). Validation of American College of Radiology Ovarian-Adnexal Reporting and Data System Ultrasound (O-RADS US): analysis on 1054 adnexal masses. Gynecol Oncol.

[30] Van Calster B, Van Hoorde K, Valentin L, Testa AC, Fischerova D, Van Holsbeke C (2014). Evaluating the risk of ovarian cancer before surgery using the ADNEX model to differentiate between benign, borderline, early and advanced stage invasive, and secondary metastatic tumours: prospective multicentre diagnostic study. BMJ (Clinical Res ed).

[31] Hsu ST, Su YJ, Hung CH, Chen MJ, Lu CH, Kuo CE (2022). Automatic ovarian tumors recognition system based on ensemble convolutional neural network with ultrasound imaging. BMC Med Inf Decis Mak.

[32] Charkhchi P, Cybulski C, Gronwald J, Wong FO, Narod SA, Akbari MR (2020). CA125 and ovarian Cancer: a Comprehensive Review. Cancers.

[33] Kisielewski R, Tołwińska A, Mazurek A, Laudański P (2013). Inflammation and ovarian cancer–current views. Ginekologia Polska.

[34] Chiappa V, Bogani G, Interlenghi M, Salvatore C, Bertolina F, Sarpietro G (2021). The adoption of Radiomics and machine learning improves the diagnostic processes of women with ovarian MAsses (the AROMA pilot study). J Ultrasound.

[35] Chiappa V, Interlenghi M, Bogani G, Salvatore C, Bertolina F, Sarpietro G (2021). A decision support system based on radiomics and machine learning to predict the risk of malignancy of ovarian masses from transvaginal ultrasonography and serum CA-125. Eur Radiol Experimental.

[36] Li S, Liu J, Xiong Y, Pang P, Lei P, Zou H (2021). A radiomics approach for automated diagnosis of ovarian neoplasm malignancy in computed tomography. Sci Rep.

[37] Russakovsky O, Deng J, Su H, Krause J, Satheesh S, Ma S (2014). ImageNet large scale visual recognition challenge. Int J Comput Vis.

[38] Gong J, Zhang W, Huang W, Liao Y, Yin Y, Shi M (2022). CT-based radiomics nomogram may predict local recurrence-free survival in esophageal cancer patients receiving definitive chemoradiation or radiotherapy: a multicenter study. Radiotherapy Oncology: J Eur Soc Therapeutic Radiol Oncol.

[39] Zheng YM, Che JY, Yuan MG, Wu ZJ, Pang J, Zhou RZ (2023). A CT-Based Deep Learning Radiomics Nomogram to predict histological grades of Head and Neck squamous cell carcinoma. Acad Radiol.

[40] Zeng Q, Li H, Zhu Y, Feng Z, Shu X, Wu A (2022). Development and validation of a predictive model combining clinical, radiomics, and deep transfer learning features for lymph node metastasis in early gastric cancer. Front Med.

