# Supplementary material for: Ultrasound-based deep learning radiomics model for differentiating benign, borderline, and malignant ovarian tumours: a multi-class classification exploratory study
Source: BMC Med Imaging. 2024 Apr 15;24:89. doi: 10.1186/s12880-024-01251-2 (PMC11020982; doi:10.1186/s12880-024-01251-2)
Supplement: Supplementary file 1 — Supplementary Material 1 [file 12880_2024_1251_MOESM1_ESM.pdf]

Cosine decay learning rate algorithm

$$\eta_t^{task-spec} = \eta_{min}^i + \frac{1}{2}(\eta_{max}^i - \eta_{min}^i)\left(1 + cos\left(\frac{T_{cur}}{T_i}\pi\right)\right) \quad (1)$$

$\eta_{min}^i = 0$, η_{max}^i =0.01, $T_i = 50$ represents the minimum learning rate, the maximum learning rate, and the number of iteration epochs, respectively. The backbone part adopts pretraining parameters. To ensure the migration effect on $T_{cur} = \frac{1}{2}T_i$ Fine-tune the parameters of the backbone part. The learning rate of the backbone part is as follows:

$$\eta_t^{backbone} = \begin{cases} 0 & \text{if } T_{cur} \leq \frac{1}{2}T_i \\ \eta_{min}^i + \frac{1}{2}(\eta_{max}^i - \eta_{min}^i)\left(1 + cos\left(\frac{T_{cur}}{T_i}\pi\right)\right) & \text{if } T_{cur} > \frac{1}{2}T_i \end{cases} \quad (2)$$

Other hyperparameter configurations are optimizer: SGD, loss function: sigmoid cross entropy.
